# Observing micro-evolutionary processes of viral populations at multiple scales

**DOI:** 10.1098/rstb.2012.0203

**Published:** 2013-03-19

**Authors:** Richard J. Orton, Caroline F. Wright, Marco J. Morelli, Nicholas Juleff, Gaël Thébaud, Nick J. Knowles, Begoña Valdazo-González, David J. Paton, Donald P. King, Daniel T. Haydon

**Affiliations:** 1 College of Medical, Veterinary and Life Sciences, Institute of Biodiversity, Animal Health and Comparative Medicine, University of Glasgow, Glasgow G12 8QQ, UK; 2 Institute for Animal Health, Ash Road, Pirbright GU24 0NF, UK; 3 Center for Genomic Science of IIT@SEMM, Istituto Italiano di Tecnologia at the IFOM-IEO Campus, Via Adamello 16, Milano 20139, Italy; 4 INRA, UMR BGPI, Montpellier Cedex 5 34398, France

**Keywords:** virus, evolution, scales, foot-and-mouth disease, transmission, bottlenecks

## Abstract

Advances in sequencing technology coupled with new integrative approaches to data analysis provide a potentially transformative opportunity to use pathogen genome data to advance our understanding of transmission. However, to maximize the insights such genetic data can provide, we need to understand more about how the microevolution of pathogens is observed at different scales of biological organization. Here, we examine the evolutionary processes in foot-and-mouth disease virus observed at different scales, ranging from the tissue, animal, herd and region. At each scale, we observe analogous processes of population expansion, mutation and selection resulting in the accumulation of mutations over increasing time scales. While the current data are limited, rates of nucleotide substitution appear to be faster over individual-to-individual transmission events compared with those observed at a within-individual scale suggesting that viral population bottlenecks between individuals facilitate the fixation of polymorphisms. Longer-term rates of nucleotide substitution were found to be equivalent in individual-to-individual transmission compared with herd-to-herd transmission indicating that viral diversification at the herd level is not retained at a regional scale.

## 1. Introduction

Foot-and-mouth disease virus (FMDV) is a non-enveloped, positive-sense, single-stranded RNA virus in the *Aphthovirus* genus of the family *Picornaviridae*. RNA viruses such as FMDV evolve rapidly owing to their large population size, high replication rate and the poor proof-reading ability of their RNA-dependent RNA polymerase. The mutation rates of RNA viruses are variously cited to be between 10^−3^ and 10^−6^ mutations per nucleotide per transcription cycle [1–4]. As a result, RNA viruses exist within their hosts as complex, heterogeneous populations, comprising non-identical genome sequences [5–7].

An integral part of any disease control strategy is the epidemiological reconstruction of virus transmission pathways, conducted by tracing the past movements of infected individuals and identifying transmission events between infected and susceptible individuals. Over the past decade, molecular and phylogenetic methods have been used increasingly for tracing and verifying FMDV transmission pathways [8–16]. These methods use genetic data, such as full or partial genome sequences, and take advantage of the virus's inherent capacity to evolve quickly to identify transmission pathways based on shared mutations. Global tracing of FMDV movements has been successfully achieved using VP1 sequences, which encode one of the three surface exposed capsid proteins of the virus [8,9,15]. However, at shorter ‘epidemic’ time scales, where the viral populations have not substantially diverged, VP1 sequencing cannot provide the required resolution. At this scale, complete genome sequencing has been proved to be a useful tool for transmission tracing [10–14,16].

Complete genome sequencing is typically performed on the whole viral sample and therefore only identifies the consensus sequence within the sample, masking the complex substructure of minority variants present. Thus, the level of resolution afforded by consensus sequencing cannot uncover all the processes underlying virus evolution at the intra- and inter-host scales. As a consequence, how variability is generated within the host and transmitted on to the next host is still poorly understood, and this impedes our ability to extract robust detailed epidemiological inferences from consensus sequence data. Although it is possible to study within-host diversity using Sanger methods by undertaking serial dilution, followed by cloning and then sequencing multiple clones [17], this is laborious and time consuming. Recent next generation sequencing (NGS) techniques provide the means for rapid and cost-effective dissection of viral evolutionary dynamics at an unprecedented level of detail [18–26]. The resolution and high throughput nature of NGS platforms have the potential to allow differentiation between samples at the inter- and intra-host scale of infection. NGS techniques have already been applied to compare ‘longitudinal’ samples of hepatitis C virus (HCV), human immunodeficiency virus (HIV) infection/transmission [27–30], as well as FMDV itself [25,26].

**Figure 1.**
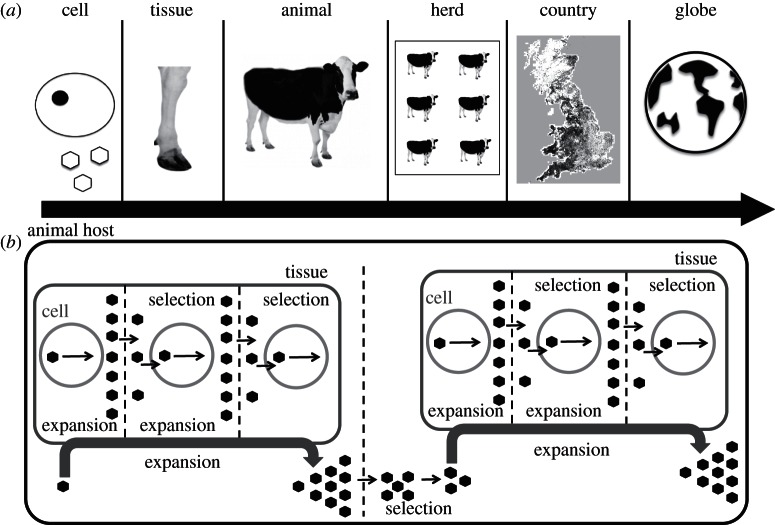
Multiple scales at which FMDV evolution can be observed. (*a*) Virions containing the FMDV genome infect cells, where all viral replication occurs; higher scales at which the evolutionary process can be observed are the tissue (a set of cells), the host animal (a set of tissues), the herd (a set of animals), a country (a set of herds) and the globe. (*b*) The fundamental processes of population expansion, transmission and selection, which occur at each scale, illustrated for the cell and tissue scales.

The evolution of FMDV can be observed at a number of distinct biological scales [31], for example the cell, tissue, host, herd, country, continent and inter-continental (figure 1*a*). Perhaps, the most commonly encountered scale is that of the individual host as it is at this scale that FMDV is detected and that the majority of data are available. However, virus samples are usually collected from a particular tissue from within an individual—for example from fluid or epithelium from vesicles on a single foot, from vesicles in/around the mouth or from oesophageal–pharyngeal scrapings (known as probang samples), and it is evident that these different populations can themselves become differentiated through drift or selection for tissue-specific tropisms. Each tissue within an animal is itself comprised cells, and it is already possible to examine micro-evolutionary processes occurring at the scale of individual cells [32,33].

There is an obvious sequence of scales moving up from that of the individual. FMDV is commonly managed at a herd scale. However, the majority of data at the herd scale comes from the scale below—the individual animal—as typically samples are only obtained from a single animal (often the animal with the oldest lesion) and used to represent the herd in molecular phylogenetic methods that generate herd-to-herd transmission trees during an epidemic [10–12]. Moving to larger scales from the herd, one might recognize a geographical region such as *County*, then a *Country*, a *Continent* and the world as a whole. The diversity of FMDV observed at any given scale can be related to that observed at the scale directly below, which is in turn a function of the scale below that: a county is a set of herds, a herd a set of animals, an animal a set of tissues and a tissue a set of cells. The smallest scale that we consider here is that of the cell as this is where all viral replication occurs and is therefore the building block for all higher scales.

The same fundamental processes operate as virus spreads between units within any given scale. First, there is population expansion, whereby virus enters a unit (be it a cell, tissue, animal or herd) and replicates from the founding inoculum. Second, a subset of this population is then transmitted on to a subsequent unit. Selection may take place during both the population expansion process (e.g. viruses that replicate fastest within the unit will be favoured) and during the transmission of virus to the next unit (e.g. viruses that can enter the unit earliest will be favoured). For example, FMDV enters and infects a cell, the viral population then expands within the cell (accumulating mutations) to the point of cell lysis and virions are released into the local environment. A sample of the progeny from the first cell then enters and infects a second cell. This sample may be small relative to the total virion output of the first cell reflecting a transmission bottleneck. Furthermore, the sample may or may not be a random sample of the amplified progeny as the result of the action of selection. This may then be repeated for multiple generations as cells repeatedly infect other cells within a tissue with mutations in the genome potentially becoming fixed or drifting in frequency over time. FMDV replication dynamics at the scale of single cells has not been studied empirically, although this would now be possible. The process has been modelled mathematically generating a number of possible predictions [34,35].

To date, there has been no direct comparison of the sequence data, diversity and substitution rates that are observed at each of these different scales, and we proceed by presenting an overview of the sequence data, comparable evolutionary metrics and summary statistics that can be observed at each of these different scales.

## 2. Methods

We now provide an overview of the sequence data and analysis techniques available at each of the scales considered for this analysis, specifically the tissue, animal and herd.

### (a) Mutation spectrum

We use the mutation spectrum [25] to characterize the heterogeneity in a viral population from NGS sequence data. The spectrum is generated by grouping nucleotide sites in the FMDV genome into discrete bins based on their observed polymorphic frequencies, and then plotting the proportion of nucleotide sites in each polymorphic bin (*y*-axis) against the polymorphic (mismatch) frequency of the bin (*x*-axis) on a log–log plot. Polymorphic frequencies for each site are calculated with reference to the inoculum used, which makes the mutation spectrum unfolded. This spectrum provides a richer view of the diversity within a viral population, and enables comparison between populations.

### (b) Mutation and substitution rate

We refer to the actual error rate of the polymerase as the mutation rate (mutations per nucleotide per transcription cycle). The rate of nucleotide substitution in a DNA sequence is defined as the number of nucleotide substitutions observed to occur per nucleotide site per unit time. Substitution rates for transmission chains were calculated using the software package Beast [36]. A variety of molecular clocks (strict, exponential relaxed) and population growth (constant, exponential) models were evaluated and compared, all of which used the HKY model (as used in previous FMDV analyses; [11]) of base substitution with the gamma model of site heterogeneity. Tip dates were assigned based on either the date the sample was taken during a real epidemic or the number of days that had passed since the start of the experiment to the sample date for the serial cow-to-cow infection studies.

### (c) Tissue

For the tissue scale, we use sequence data from a study that used NGS technology to analyse the viral population within a foot lesion on a single animal [25]. Briefly, a single bovine host was inoculated with FMDV and 2 days post-inoculation, a sample was taken from an FMD epithelial lesion that developed on the front left foot.

### (d) Animal

For the animal scale, we use the consensus sequences generated from samples taken at various points during the infection of a single animal, specifically animal number two in [26]; data available from the EBI SRA repository (http://www.ebi.ac.uk/ena/) accession number ERP001880 from 1 May 2013. Briefly, a calf was naturally challenged by direct contact with another infected calf. A total of nine samples were then collected from this second calf at a range of days post first contact (DPFC) from different tissues. The samples were processed and sequenced on an Illumina platform in the same way as the *Tissue* scale described earlier. Consensus sequences for each sample were then generated from the reads aligned to the reference genome. A genealogy of the samples within the animal can then be created based on statistical parsimony analysis of the consensus sequences using the software package TCS [37].

### (e) Herd

For the herd scale, we use and compare sequence data from two types of dataset. First, serial cow-to-cow infection chains from controlled experiments from [38] where the consensus sequences from viral samples were generated using Sanger sequencing for each animal in two independent cow-to-cow infection chains, one consisting of four animal hosts and the other six; some animals had multiple consensus sequences generated from different samples taken at varying DPFC. Second, we use data from herd-to-herd transmission chains inferred from consensus sequence analysis from 2001 to 2007 FMD epidemics in Great Britain (GB). Briefly, information from consensus sequences was combined with dates of disease detection and lesion age to generate the most probably transmission trees between herds during the 2007 epidemic [12] and a cluster within the 2001 epidemic [11]. As above, TCS was used to generate genealogical relationships among sequences.

## 3. Results

### (a) Tissue

**Figure 2.**
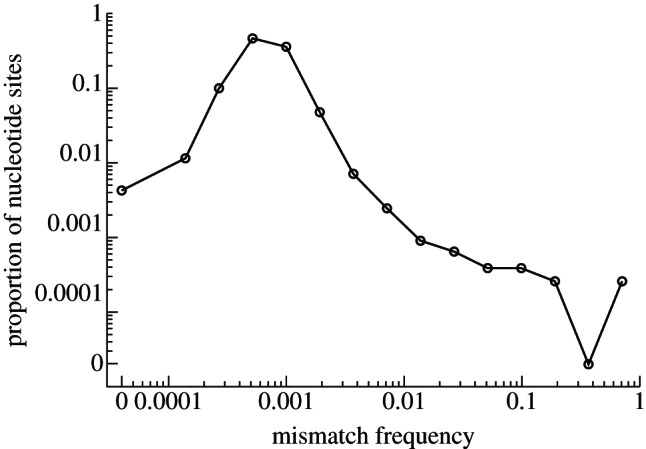
Mutation spectrum of a lesion. The black line represents the mutation spectrum generated from NGS sequence data from a cow foot lesion.

The mutation spectrum of a lesion (figure 2) shows that the majority of nucleotides display low-frequency polymorphisms. However, higher-frequency mutations are observed, including a number at the consensus (100%) level. Wright *et al.* [25] used the presence of stop codons in the lesion sequence data to obtain an upper limit on the mutation rate of the virus under the hypothesis that such mutations are lethal, and were therefore generated in the last round of cellular replication. An upper bound of 7.8 × 10^−4^ mutations per nucleotide per transcription event was estimated (95% CI: 7.4–8.3 × 10^−4^) in line with previous estimates [2,39,40]. In related work, Cottam *et al.* [17] sequenced the capsid region of the FMDV genome of 26 clones created from the same cow epithelium viral sample and observed a mutation frequency of 2.79 × 10^−4^ mutations per nucleotide sequenced providing further insight into the population diversity that exists within a single lesion.

### (b) Animal

**Figure 3.**
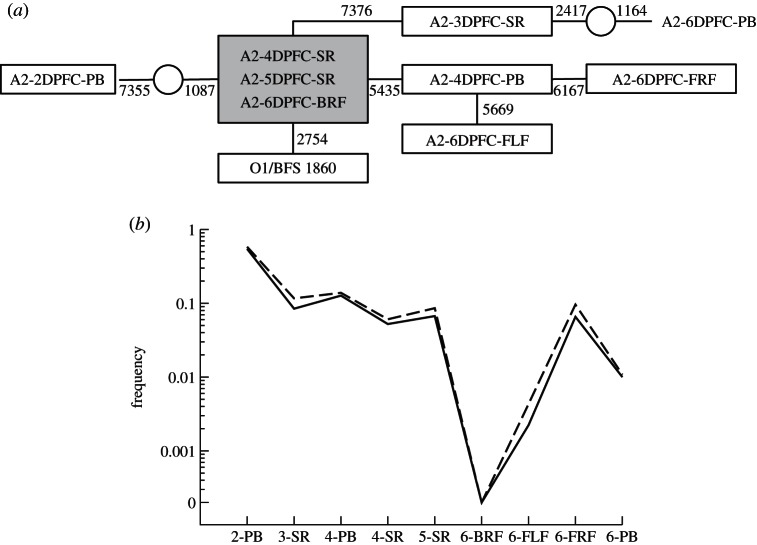
Genetic network of intra-host tissue samples. (*a*) Genealogy of nine samples from cow number 2 in the cow-to-cow infection chain in [26]. A consensus sequence was generated for each sample from the NGS data and a statistical parsimony tree using the software package TCS [37]. Samples are labelled according to the animal number (A2), followed by the number of DPFC, and then the tissue type (probang, PB; serum, SR; or foot: BRF, back right foot; FLF, front left foot; FRF, front right foot). The original O1/BFS 1860 FMDV inoculum is also shown in tree. Samples located within the same box share the same sequence, links between boxes represent single mutations, with additional unsampled genomes represented with open circles; the genome position at which changes distinguish the different genotypes is indicated next to each link. The box shaded in grey represents the animals overall consensus sequence. (*b*) Mutation frequency (*y*-axis) of genome positions 1087 (straight line) and 7355 (dotted line) across all samples; the bottom *x*-axis represents the number of DPFC followed by the sample type.

There is substantial viral diversity within a single cow, even when examining tissue-specific consensus sequences (figure 3*a*). Distinct within-host lineages are evident, for example, the three feet samples all have different consensus sequences even though they were all obtained 6 DPFC; furthermore, all of the feet are also different to the probang sample taken on the same day. There are also consensus level mutations appearing in only a single sample, for example the probang sample on day 2 acquired two consensus level mutations at genome positions 1087 and 7355, which are not observed at the consensus level in any other sample. Overall, figure 3*a* highlights the complexity of the intra-host dynamics of FMDV evolution. An animal's consensus sequence can be generated by aligning the consensus sequences of all the individual samples and identifying the most common base at each nucleotide site. In this case, the consensus sequence of the animal itself contains only a single mutation from the original inoculum at genome position 2754. All the individual samples from this animal contain the 2754 mutation and are between 0 and 3 mutations away from the animal-level consensus. Therefore, as this mutation is shared across all tissues and time points within the animal, it will very likely be passed on to the next host, enabling reconstruction of transmission trees based on shared mutations.

As the underlying sequence dataset is generated by NGS approaches, it is also possible to monitor sub-consensus mutations at the within-host level by tracking the polymorphic frequency of a particular nucleotide through each of the samples. For example, figure 3*b* shows the mutation frequencies of bases 1087 and 7355 across all nine samples. These two mutations were observed at the consensus level in the probang sample after 2 days but not observed in any other sample—at the tissue-specific consensus level. These mutations do not simply disappear but are present in all other samples at sub-consensus levels, gradually decreasing in frequency over time (figure 3*b*).

### (c) Herd

**Figure 4.**
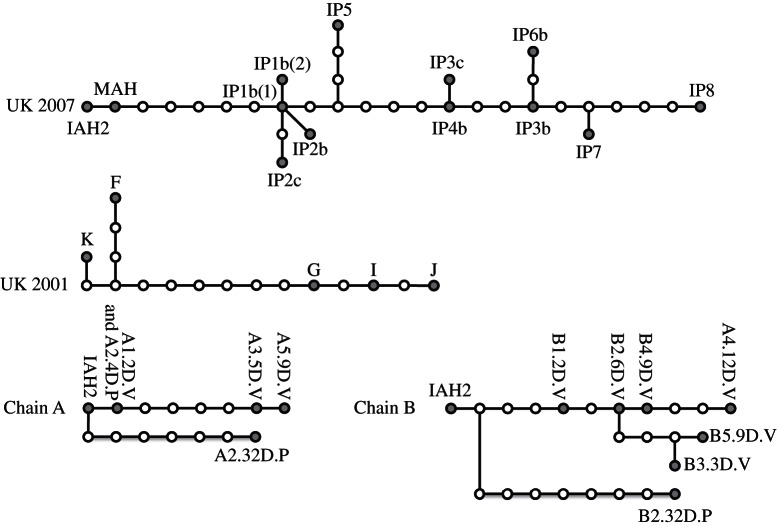
Statistical parsimony trees of FMD transmission between cows and herds: UK2007 and UK2001 represent the between-herd transmission tree from the full 2007 FMD epidemic [12] and the largest chain from the 2001 epidemic [11], respectively. Chain A and Chain B represent two between cow transmission chains from the study of Juleff *et al.* [38]. In all cases, solid black circles represent samples from different cows or herds, open circles represent unsampled genomes and the connecting lines represent single nucleotide differences between genomes.

**Figure 5.**
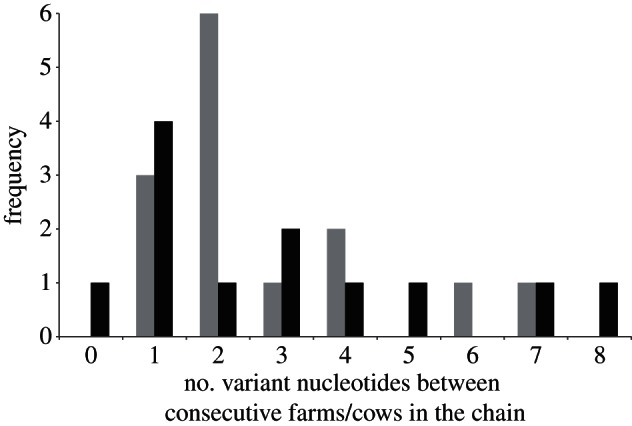
Distribution of the number of variant nucleotides between viruses recovered from consecutively infected herds and cows. The number of variant nucleotides between herds (grey) and cows (black) was calculated from their respective herd-to-herd and cow-to-cow transmission chains. The number of variant nucleotides was determined from the common ancestor of source and daughter herd if not directly linked.

At the herd scale we compare two types of data: (i) serial cow-to-cow infection chains from controlled experiments and (ii) serial herd-to-herd infection chains from real FMD epidemics in the UK. Experimentally manipulated serial cow-to-cow infection chains are not herds because, although they are a set of animals, their transmission is restricted to a serial one-to-one sequence of transmission unlike the one-to-many relationship that can occur when an infected individual enters a susceptible herd. However, we use them here to investigate whether there is a difference between rates of evolution observed across cow-to-cow and herd-to-herd transmission events. Figure 4 displays the statistical parsimony trees depicting the genetic relationship between viruses from two cow-to-cow infection chains from the study of Juleff *et al.* [38] and two herd-to-herd infection chains from the studies of Cottam *et al.* [11,12] representing the GB 2001 and 2007 epidemics. We can compare rates of evolution observed at cow–cow and herd–herd transmission scales by comparing the distribution of the number of nucleotide changes per herd and per cow in their respective transmission chains. Figure 5 shows that the two distributions are not statistically distinguishable, with an average of 2.78 mutations between herds and 3.00 between cows (Wilcoxon rank sum test *W* = 106, *p* = 0.815). This suggests that there is little difference in the rate at which mutations accumulate as a result of herd-to-herd and cow-to-cow transmission events. Previous estimates of the substitutions per genome per herd-to-herd transmission range from 1.5 [10] to 4.3 [11] for the 2001 UK FMD epidemic.

Table 1 presents an overview of the substitution rates calculated using Beast [36] for the two cow-to-cow transmission chains, the herd-to-herd chain from the GB 2007 FMD epidemic, along with previously published estimates from herd-to-herd transmission chains from the GB 2001 FMD epidemic. The substitution rates for all the chains are very similar, with overlapping confidence intervals, again suggesting little difference between herd-to-herd and cow-to-cow transmission events.

**Table 1.** Comparison of substitution rates between transmission chains. Strict, strict molecular clock; relaxed, exponential relaxed molecular clock; constant, constant size; exponential, exponential growth; 95% CIs shown in brackets.

dataset	molecular clock model	coalescent model	marginal mean log likelihood	substitution rate (×10^−5^ mutations per nucleotide per day)
cow-to-cow (chain A)	strict	constant	−11493.09	2.27 (0.770–3.90)
	strict	exponential	−11492.27	2.26 (0.728–3.91)
	relaxed	exponential	−11493.55	1.94 (0.205–3.78)
	relaxed	constant	−11494.03	1.97 (0.313–3.76)
cow-to-cow (chain B)	strict	constant	−11605.52	2.86 (1.32–4.43)
	strict	exponential	−11603.95	2.91 (1.41–4.57)
	relaxed	exponential	−11598.06	3.21 (1.42–5.28)
	relaxed	constant	−11600.19	3.05 (1.15–4.95)
herd-to-herd (2007)	strict	constant	−11650.38	2.51 (1.43–3.74)
	strict	exponential	−11647.06	2.61 (1.45–3.87)
	relaxed	exponential	−11640.31	3.09 (1.59–4.82)
	relaxed	constant	−11644.21	2.97 (1.48–4.65)
herd-to-herd (2001)^a^	relaxed	exponential		2.26 (1.75–2.8)
herd-to-herd (2001)^b^	relaxed	constant		2.08 (0.574–3.51)

^a^Adapted from [10].

^b^Adapted from [11].

**Figure 6.**
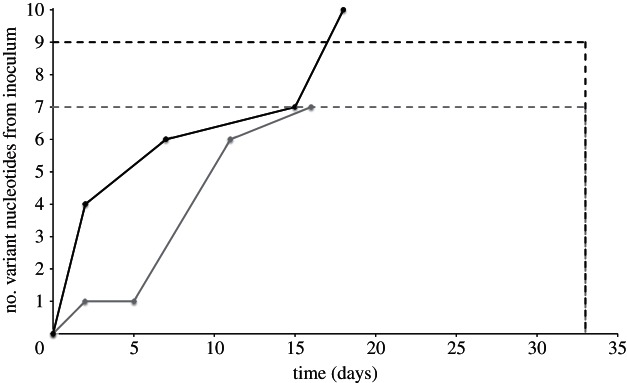
Comparison of within and between nucleotide variations. The solid grey and black lines represent the accumulated number of variant nucleotides over time between consensus sequences along the A1 → A5 and B1 → B5 chains (figure 5), respectively. The dashed grey (A chain) and black (B chain) lines represent the number of variant nucleotides observed in animal number 2 of the chain in the sample taken 32 DPFC.

We can compare the rates of evolution measured over the same time periods both within a cow and as virus is passed between cows. In both A and B cow-to-cow chains, a probang sample from animal number 2 was taken 32 DPFC. Figure 6 shows that as the virus is transmitted between animals, it appears to evolve faster compared with when the virus is confined to a single host, reaching the same number of consensus mutations but in approximately half the time. This suggests that cow-to-cow transmission events may be important in determining the substitution rate of the virus. This slower rate within a host could be due to the host immune system, with the within-host evolution rate slowing down over the course of infection due to a reduction in the volume of viral replication within the host over time. Alternatively, tight bottlenecks between hosts could result in a higher rate of substitutions observed in consensus level sequences. As the viral population in the infected animal is very diverse, there is a high probability that the virions transmitted to the next animal contain mutations, and the smaller the bottleneck, the higher the proportion of infections starting from mutated variants only.

## 4. Discussion

We have introduced a ‘multi-scale’ approach for observing viral evolution using sequence data enabling the viral diversity observed at any given scale to be related to that observed at the scale directly below. Irrespective of the scale being observed, the same fundamental processes operate—population expansion, transmission and selection—and we observe these processes at increasing temporal and spatial scales moving up from the single transcription event, which actually introduces the mutations into the genome, to tissue, individual, herd and regional scales. Through our analyses, we observed similar rates of evolution between herds and between individuals suggesting there is little difference between cow-to-cow and herd-to-herd transmission. However, we observed faster rates of evolution between hosts compared with within hosts, suggesting bottlenecks or the host immune system could be the important factors influencing the observed rate of substitution at the within-host level.

Faster rates of evolution between hosts could be explained by tight cow-to-cow transmission bottlenecks. Given the high probability that virions transmitted to the next cow contain mutations (due to the diverse viral population of the infected cow), a tight bottleneck could result in a higher proportion of infections starting from mutated virions only in the next cow. As a result, a higher fixation rate in consensus level sequences will be observed, as the virus moves from host to host. The host immune system could also be an important factor, with the within-host evolution rate slowing down over the course of infection owing to the host immune response reducing the volume of viral replication within the host over time.

Unlike experimentally manipulated cow-to-cow infection chains, real epidemics are rarely fully characterized. Less clinically obvious infections may be missed altogether, alternatively, control strategies such as rapid ring culling around premises infected with FMD can lead to infected herds being culled before detection, potentially leading to gaps in sequence transmission chains. Such missing herds can be thought of as epidemiological dark matter (‘dark cows’) that can be inferred from gaps in transmission chains. The development of statistical methods to estimate the number and even location of these unobserved infections remains a contemporary problem awaiting a fully satisfactory solution.

There are also difficulties with interpreting cow-to-cow infection chains. Figure 3 indicates significant variability at the within-cow level. Furthermore, Juleff *et al.* [38] noted that probang consensus sequences frequently contained ambiguities, complicating their interpretation. This could be due to the nature of such samples, which scrape the oesophageal–pharyngeal area, thus sampling from a potentially large tissue area and multiple numbers of lesions; whereas, feet samples are typically taken from a single lesion.

We see little difference between rates of substitution generated over cow-to-cow and herd-to-herd transmission events, in terms of number of nucleotide changes between units or substitution rates; however, we note the small number of datasets limits the statistical power of this inference. This could be a product of herd-to-herd transmission essentially being functionally equivalent to cow-to-cow transmission, that is to say, perhaps virus is not passaged extensively from cow-to-cow prior to transmission to a subsequent herd. Although cows do typically move in batches, it is relatively a small number that moves from farm-to-farm; Green *et al.* [41] calculated a mean batch size of three animals during the 2002–2005 period suggesting only a small sample of the viral diversity within a herd will be transmitted to the next via movements. To date, the sequence diversity of the UK epidemics at the within-herd level has not been reported, such data would enable further investigation of both within- and between-herd dynamics.

Differences between the within- and between-host evolutionary rates have previously been reported in both HCV [42] and HIV [43–45]. Gray *et al.* [42] reported higher evolutionary rates between hosts compared with within hosts for HCV, similar to our findings here. However, Gray *et al.* [42] also estimated evolutionary rates for different partitions of the HCV genome, and found substantially higher rates of evolution at within-host scale for the hyper-variable region HVR1. Future work on partitioning of the FMDV genome could lead to similar observations.

There is growing evidence that, over calendar time, HIV evolves considerably faster within individuals than it does at the between host epidemic level [43–45]. Lythgoe & Fraser [45] concluded that there is preferential transmission of ancestral virus through the cycling of virus through very long-lived memory CD4^+^ T cells, a process they termed ‘store and retrieve’. Although we observe higher evolutionary rates between hosts compared with within hosts for FMDV, there are number of factors that could account for this difference, such as our relatively small dataset, the longer time scales of HIV, the mode of transmission and the role of long-lived memory cells in HIV transmission [45].

We have focussed on FMDV due to the availability of sequence data at a variety of scales, but similar ideas can be applied to other livestock viruses such as bluetongue and Schmallenberg viruses. Similar ideas are obviously extendable to human viruses such as hepatitis C (e.g. cell, organ, person and city) and plant viruses (e.g. cell, leaf (tissue), tree and orchard) such as the *Plum pox* virus L395 which is a serious viral disease of stone fruit, transmitted by aphids, which causes acidities and deformities in the fruit [46].
